# Castration-induced testosterone deficiency increases fasting glucose associated with hepatic and extra-hepatic insulin resistance in adult male rats

**DOI:** 10.1186/1477-7827-11-106

**Published:** 2013-11-18

**Authors:** Fangzhen Xia, Xiao Xu, Hualing Zhai, Ying Meng, Huixin Zhang, Shichun Du, Hui Xu, Hui Wu, Yingli Lu

**Affiliations:** 1 Endocrinology and Metabolism Research Institute and Department of Endocrinology and Metabolism, Shanghai Ninth People’s Hospital Affiliated to Shanghai Jiaotong University School of Medicine, Shanghai 200011, China

**Keywords:** Orchidectomy, Metabolic kinetics, Isotopic trace, Insulin resistance, Hyperinsulinemic-euglycemic clamp

## Abstract

**Background:**

Testosterone deficiency is associated with insulin resistance. However, how testosterone deficiency affects insulin actions remains unclear. The aim of this study was to investigate the influence of castration-induced testosterone deficiency on the metabolic kinetics of glucose and to evaluate the hepatic and extra-hepatic insulin sensitivity, in advanced-age male Sprague–Dawley (SD) rats.

**Methods:**

Ten-week-old male SD rats were randomly divided into three groups: (1) a control group (n = 10) in which the rats underwent sham castration (2) a castrated group (TD group for testosterone deficiency, n = 10) in which the rats underwent bilateral orchidectomy surgery and (3) a castrated group given testosterone propionate via intraperitoneal injection (25 mg/kg/day) to supplement androgen (TD + TP group, n = 10). At ten weeks after castration in the noted groups, all rats were subjected to an oral glucose tolerance test (OGTT), a pyruvate tolerance test (PTT) and an insulin tolerance test (ITT). Twenty weeks following that treatment, all rats underwent a hyperinsulinemic-euglycemic clamp procedure in conjunction with isotope--labeled glucose and glycerol tracer infusions. The rate of appearance (Ra) of glucose, glycerol and gluconeogenesis (GNG), hepatic glucose production and the rate of glucose disappearance (Rd) were assessed. Glucose uptake was determined by measuring the 2-deoxy-D-14C-glucose in the gastrocnemius muscles.

**Results:**

Ten weeks after castration in the TD group, the fasting blood glucose and insulin levels were significantly increased (p < 0.01), the glucose-- induced insulin secretion was impaired and ITT revealed a temporarily increased whole body insulin sensitivity compared with the control group; 30 weeks after castration, the Ra of glucose, Ra of glycerol, as well as the HGP and GNG were also increased (p < 0.01), while the exogenous glucose infusion rate and uptake glucose in the muscle markedly decreased (p < 0.01).

**Conclusions:**

Castration-induced testosterone deficiency primarily increases fasting blood glucose levels. The clamp experiments revealed a clear insulin resistance both at the hepatic and extra-hepatic levels.

## Background

Late-onset hypogonadism (LOH) or age-associated testosterone deficiency syndrome, which is defined as a clinical and biochemical syndrome associated with advancing age and is characterized by symptoms of hypogonadism and a deficiency in serum testosterone levels, has become a world-wide concern because it adversely affects the function of multiple organ systems [1]. Patients with LOH not only have the symptoms of testosterone deficiency, but the incidences of metabolic syndrome have also notably increased [2]. Epidemiological studies have shown a direct correlation between plasma testosterone and insulin sensitivity, and low testosterone levels are associated with an increased risk of type-2 diabetes mellitus [3]. Prospective studies indicated that the risk of type 2 diabetes can be reduced by 42% in men with normal testosterone levels [4]. However, clinical intervention studies reported controversial results regarding the effects that testosterone replacement therapy has on insulin resistance in aging males [5]. This controversy is partly due to the lack of long-term safety and efficacy of testosterone replacement therapy. Additionally, the mechanism of how testosterone affects insulin actions remains unclear. Morimoto S et al. [6] found that testosterone has a direct effect upon pancreatic islet function by favoring insulin gene expression and release. Christoffersen B et al. [7] observed that castration-induced testosterone deficiency in male rats led to insulin resistance; however, they only describe the parameters related to the metabolic syndrome. The mechanism of how testosterone deficiency affects insulin actions and the metabolic kinetics of glucose has not yet been identified to date.

Insulin regulates glucose homeostasis through binding to the extra cellular domain of the insulin receptor in its responsive target organs such as skeletal muscles, liver and adipose tissue. When homeostasis is reached, the rate of glucose appearance (Ra) is equal to that of glucose disappearance (Rd). Endogenous glucose production (EPG) in the liver and glucose uptakes by skeletal muscles and adipose tissue are crucial factors that contribute to the homeostasis of glucose. Glucose uptake by skeletal muscle accounts for 75 to 80% of normal insulin-stimulated whole-body glucose disposal after a meal [8]. Hepatic or extra-hepatic insulin resistance leads to abnormal EPG, Rd or uptake, which can induce instability in glucose homeostasis. There is no data demonstrating how testosterone deficiency affects these parameters. This study aimed to investigate the influence of testosterone deficiency on glucose metabolism kinetics using the isotope technique in combination with hyperinsulinemic euglycemic clamps in castrated advanced-age male rats.

## Methods

### Experimental animals

All animal experiments were approved by the Animal Experimental Ethical Committee, the Ninth People’s Hospital, School of Medicine, Jiao Tong University, Shanghai, China. The approval data was June 1, 2012 and the Reference Number was HKDL [2012] 6.

**Figure 1:**
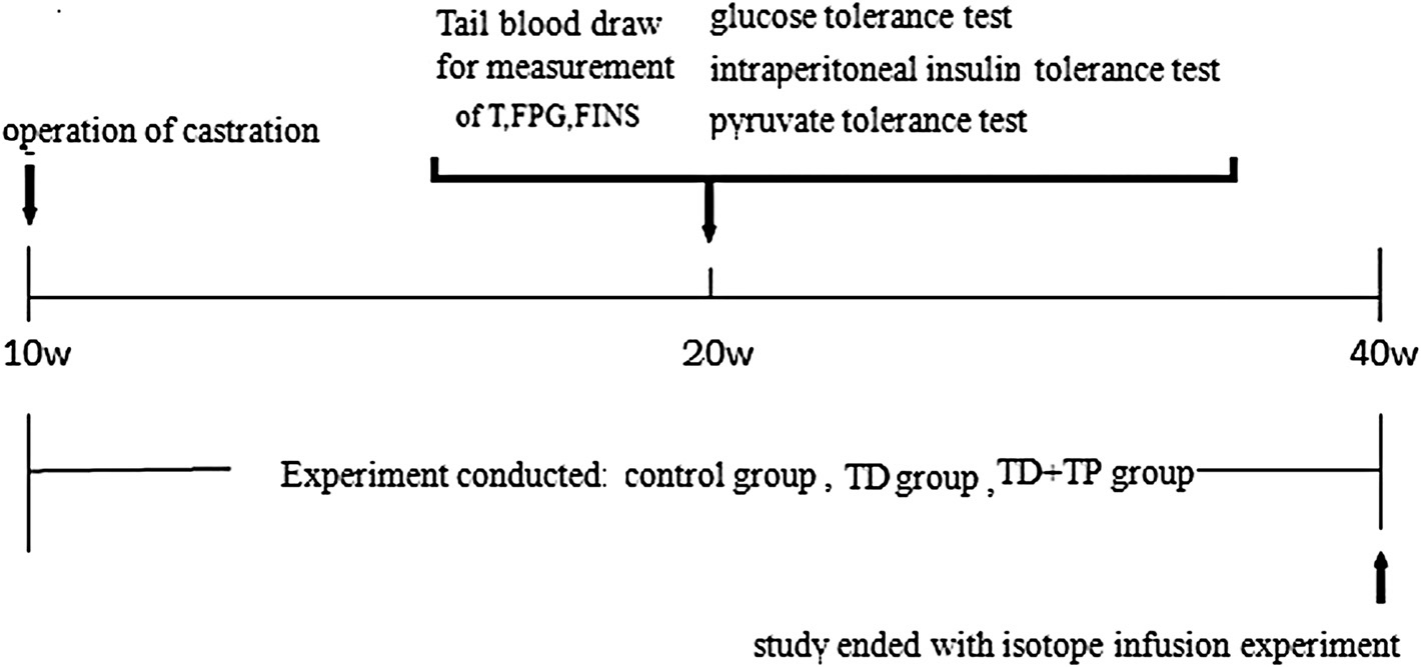
Research protocol of the experiment.

The study comprised 30 male Sprague–Dawley rats (10 weeks old) that were bred and housed locally at 22+/−2°C under a 12 h on/12 h off light cycle with free access to food and water. The experiments were performed in conformity with the Guide for the care and use of laboratory animals published by the US National Institutes of Health (publication no. 85–23, revised 1996). The rats were randomly divided into three groups: (1) a control group (n = 10) in which the rats underwent sham castration (2) a castrated group (TD group for testosterone deficiency, n = 10) in which the rats underwent bilateral orchidectomy surgery and (3) a castrated group given testosterone propionate via intraperitoneal injection (25 mg/kg/day) for 30 weeks to supplement androgen (TD + TP group, n = 10). Three groups of rats were given a normal diet (52% carbohydrate, 22.1% proteins, 9.2% water, 5.28% fat, 4.12% cellulose, 4.22% mineral salts). The study ended after 30 weeks after the castration operation. (Study protocol is shown in Figure 1).

### Blood sample assays

Ten weeks after castration, tail blood samples were obtained after an overnight fast to assess glucose, insulin and testosterone in all rat groups. The plasma glucose concentrations were assayed using the Siemens Dimension MAX (Siemens Healthcare Diagnostics Inc., NY, U.S.A). The plasma insulin levels were assayed via a magnetic affinity immunoassay (Insulin MPAIA Kit, Bioekon Bio Inc., Beingjing, China). The testosterone (T) levels were assayed via a chemiluminescent Microparticle immunoassay (CMIA).

### Oral glucose tolerance test (OGTT)

All rats were fasted for 16 h before the test and received 2 g/kg glucose solution via oral gavage. Blood samples were taken by severing the tip of the tail at 0, 15, 30, 60, 90 and 120 min after glucose administration. Areas under the curves (AUC) for OGTT were calculated to evaluate glucose tolerance.

### Intraperitoneal insulin tolerance test (ITT)

The rats were fasted for 12 h before the experiments prior to IP insulin injection (0.75 IU/kg). Blood samples were collected from the tail at 0, 15, 30 and 60 min for the serum glucose level measurements. Areas under the curve for ITT were calculated to evaluate insulin sensitivity.

### Pyruvate tolerant test (PTT)

The rats were fasted for 16 h before the test, and a sodium pyruvate solution (1 g/kg) was IP-injected. The glucose levels were determined in the blood collected from the tail at 0, 15, 30, 60, 90 and 120 min after the pyruvate injection. The areas under the curve of glycemia vs. time were calculated to estimate the total glucose synthesized from pyruvate.

### Isotope infusion procedure

**Figure 2:**
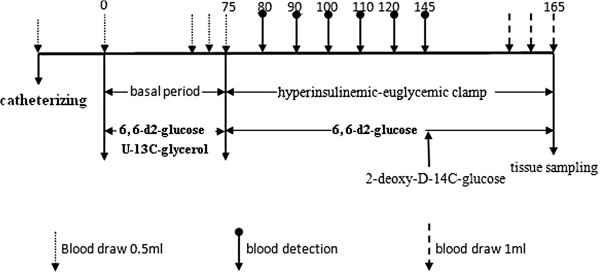
Schematic diagram of the study tracer infusion protocol.

Thirty weeks after castration, the rats were fasted overnight and underwent isotope infusion as we previously reported [9]. The tail artery was catheterized for blood collection and established an optimal V-A mode of metabolic experiments. The procedure is shown in Figure 2. Blood samples were collected at 0, 65 and 70 min.

### Hyperinsulinemic euglycemic clamp

The appearance rates of glucose were traced using [6, 6-2D] glucose, while the gluconeogenesis (GNG) rates were accessed using [U-13C]-glycerol. The hepatic glucose production (HGP) and the rate of glucose disappearance (GRd) of the fasting rats were determined using the hyperinsulinemic-euglycemic clamp with the continuous infusion of [6, 6-2D] glucose and regular insulin. The average glucose uptake rates of the gastrocnemius muscles were also determined via injection of 2-deoxy-D-14C-glucose.

The studies lasted 165 minutes and included 75 minutes of a basal clamp phase for the assessment of the basal glucose turnover and 90 minutes of a hyperinsulinemic-euglycemic clamp phase for the evaluation of insulin sensitivity. One blood sample was obtained immediately prior to starting the tracer infusion to determine the background glucose enrichment and insulin concentration in the plasma. The [6,6-2D] glucose (at a rate of 2 μmol/kg/min) and [U-13C]-glycerol (at rate of 0.84 μmol/kg/min) were then constantly infused intravenously via the IV infusion line driven using a Harvard mini infusion pump (Harvard Apparatus, Holliston, MA, U.S.A.) for 75 minutes (basal period). Blood was sampled prior to tracer infusions and at 65, 70, and 75 min. While the [6,6-2D] glucose infusion continued, a primed and continuous constant infusion of recombinant human insulin (5 mIu/kg/min, Novolin R, Novo Nordisk, Denmark) was initiated for another 90 min (chase period). The plasma glucose concentration was maintained at the basal level by monitoring the plasma glucose every 10 minutes and empirically adjusting the infusion rate of a 25% glucose solution. Twenty minutes prior to the end of the chase period, 1 μCi of 2-deoxy-D-14C-glucose (PerkinElmer, Waltham, U.S.A.) was injected through the IV infusion line for the measurement of tissue glucose uptake. During the final 10 minutes, 3 additional blood samples were collected at 3-minute intervals. A flow chart of the study design is shown in Figure 2. The rats were euthanized via cardiac puncture under anesthesia with chloral hydrate (5 mg/kg) to reduce blood elements in tissues. The gastrocnemius muscle was immediately biopsied, cut into small pieces, immersed in liquid nitrogen and stored at -80°C. The plasma samples were prepared on ice, centrifuged at 4°C, separated and stored at -80°C until further analysis.

### Gas Chromatography/Mass Spectrometry

Enrichments of the glycerol and glucose were measured using GC-MS with their trimethylsilyl derivative via methoxyamine-HCl and BSTFA to obtain m/z: 321/319 [6, 6-2D] glucose and m/z: 221/218 [U-13C]-glycerol. The GC was equipped with a DB-5MS capillary column (30 m × 0.25 mm inner diameter, 0.25 ìm film). The GC oven temperature was programmed initially at 70°C for 4 min, increased to 240°C at 10°C /min and then to 300°C at 20°C /min and sustained at 300°C for 11 min. The GC was electronically controlled for constant pressure and humidity.

### Calculation

The infusion rates (ìmol/kg/min) of [U-13C]-glycerol and [6, 6-2D] glucose were divided using the MPE of plasma glycerol and glucose, respectively, to obtain the appearance rate (ìmol/kg/min) [10].

Raglu (ìmol/kg/min) = HGP = infusion rate of [6, 6-2D] glucose (ìmol/kg/min)/ MPE of plasma glucose.

Ragly (ìmol/kg/min) = infusion rate of [U-13C]-glycerol (ìmol/kg/min)/MPE of plasma glycerol –infusion rate of [U-13C]-glycerol (ìmol/kg/min).

The gluconeogenesis rates were calculated by dividing the MPE of the plasma [U-13C]-glycerol by the MPE of the [1, 2, 3-13C] glucose. In this experiment, appreciable amounts of [U-13C]-glycerol were converted to [1, 2, 3-13C] glucose via gluconeogenesis. Thus, we could compare the gluconeogenesis rates between different groups [11].

The gluconeogenesis rates = MPE of [1, 2, 3-13C] glucose/MPE of plasma [U-13C]-glycerol.

GNG from glycerol (ìmol/kg/min) = percent of Raglu (ìmol/kg/min).

Percent of glycerol converted to glucose = Ragly (ìmol/kg/min) * MPE of [1, 2, 3-13C] glucose/infusion rate of [U-13C]-glycerol (ìmol/kg/min).

The average glucose uptake rates of the gastrocnemius muscles were calculated as follows [12]:

Glucose uptake (ìmol/g/min) = muscle 2-deoxy-D-14C-glucose radioactivity (dpm/g/min)/plasma 2-deoxy-D-14C-glucose SA (dpm/ìmol).

### Statistical analysis of data

All values were expressed as the means+/−SD. Statistical significances were evaluated using ANOVA. P values < 0.05 indicated a significant difference.

## Results

### Body weight and blood glucose levels

Prior to the bilateral orchidectomy surgery, there were no differences in body weight or random and fasting blood glucose levels between the three groups of rats (data not shown). At the completion of the study, the mean body weight in the control group was significantly higher than those in the TD group and TD + TP group. The mean body weight was also slightly higher in the TD + TP group than in the TD group, but without statistical significance (control group: 629.2+/−66.93 g, TD group: 502.86+/−66.88 g TD + TP group: 546+/−44.63 g, P = 0.0188) (Table 1).

**Table 1:** The body weight, blood glucose, testosterone and estrogen levels of the three groups of rats at the end of the study (+/−s)

	**C**	**TD**	**TD + TP**	**P value**
	**(n = 10)**	**(n = 10)**	**(n = 10)**	
*Body weight (g)*	629+/−66.93	502.86+/−66.88*	546+/−44.63*	0.0188
*RBG (mmol/L)*	4.46+/−1.08	5.36+/−0.67	4.98+/−0.438	0.1696
*FBG (mmol/L)*	4.56+/−1.173	5.514+/−0.705*^#^	3.34+/−1.171	0.0082
*T (ng/mL)*	2.96+/−0.55	1.45+/−0.26*^#^	2.07+/−0.35*	0.0053
*E2 (pg/mL)*	8.2+/−2.08	6.4+/−2.05	8.4+/−1.52	0.065

C, control group, rats underwent sham surgery; TD, testosterone deficiency group, rats underwent bilateral orchidectomy surgery; TD + TP, castrated rats given testosterone; RBG, random blood glucose; FBG, fasting blood glucose; T, testosterone; E2, estrogen; Data are presented as the means +/− SD.

*, compared with the control group (C).

#, compared with castrated rats replaced with testosterone propionate (TD + TP).

When compared with the control group and the TD + TP group, the random blood glucose levels of the TD group increased slightly (control group: 4.46+/−1.08 mmol/L, TD group: 5.36+/−0.67 mmol/L, TD + TP group: 4.98+/−0.438 mmol/L, P = 0.1696), while the fasting blood glucose levels were significantly higher (control group: 4.56+/−1.173 mmol/L, TD group: 5.514+/−0.705 mmol/L, TD + TP group: 3.34+/−1.171 mmol/L, P = 0.0082) (Table 1).

### Testosterone and estrogen concentrations

Following castration, the serum testosterone levels were significantly lower compared with those in the rat control group (TD group: 1.45+/−0.26 ng/ml, control group: 2.96+/−0.55 ng/ml, p < 0.05); testosterone propionate replacement partially raised the levels. The concentrations of estrogen were not significantly different between those in the castrated and non-castrated rats (Table 1).

### Fasting plasma insulin (FINS), Homeostasis model-Insulin resistance (HOMA-IR) and Insulin sensitivity index (ISI)

**Figure 3:**
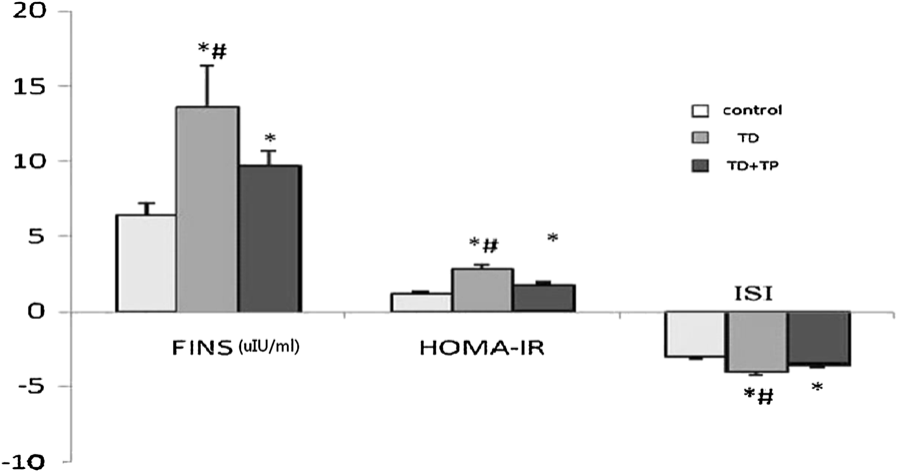
**FINS, HOMA-IR and ISI of the three groups.** Insulin concentration and HOMA-IR increased dramatically with a decrease in the insulin sensitivity index. *, compared with control group (C). ^#^, compared with castrated rats replaced with testosterone propionate (TD + TP).

The FINS concentrations in the TD group were significantly higher than those in the control group and the TD + TP group, while those in the control group significantly lower than those in the TD + TP group (control group: 6.39+/−0.83 μIu/ml, TD group: 13.61+/−2.785 μIu/ml, TD + TP group: 9.66+/−1.04 μIu/ml, P = 0.0036). The HOMA-IR, an index of insulin resistance, was significantly higher in the TD group compared with the control and the TD + TP groups (control group: 1.172+/−0.155, TD group: 2.796+/−0.299, TD + TP group: 1.745+/−0.177, P < 0.0001). The insulin sensitivity index was the opposite, as that in the TD group and TD + TP group were lower than that in the control group (control group: -3.086+/−0.122, TD group: -4.013+/−0.215, TD + TP group: -3.624+/−0.119, P = 0.0003) (Figure 3).

### The effect of testosterone deficiency on whole body insulin sensitivity

**Figure 4:**
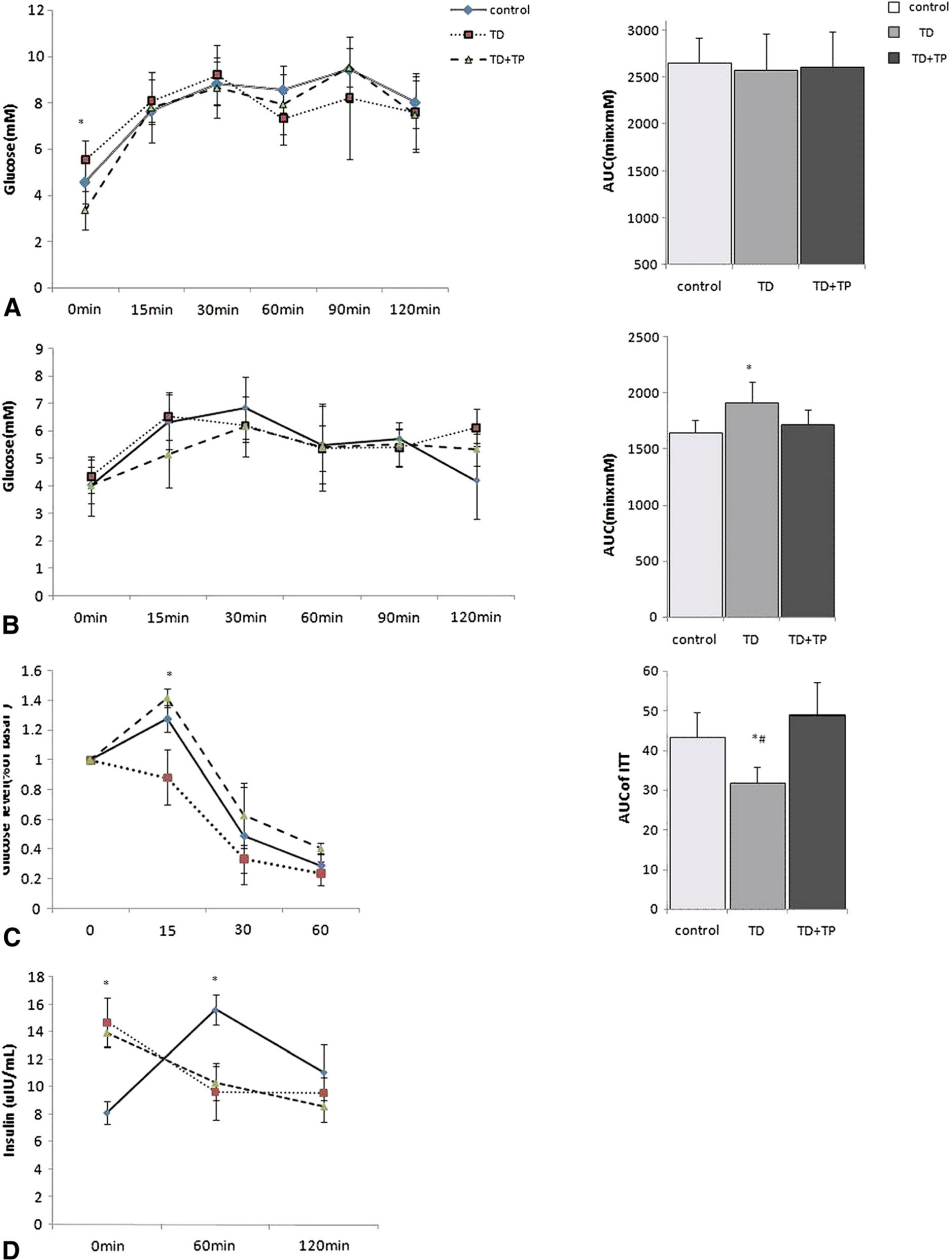
**OGTT, ITT and PTT of the three groups.** Oral glucose tolerance test and AUC of OGTT **(A)**, Pyruvate tolerance test and AUC of PTT **(B)**, Insulin tolerance test and AUC of ITT **(C)**, Glucose-stimulated insulin secretion **(D)**. *, P <0.05 compared with the control group. #, P <0.05 compared with the TD + TP group.

Ten weeks after the surgery, the fasting glucose levels in the TD group were slightly and not significantly higher compared with controls. An analysis of the OGTT revealed that castration had no significant effect on glucose intolerance following glucose challenge and that the AUCs of OGTT in all groups were similar (Figure 4A). The rats in the TD group exhibited significantly lower serum insulin levels in response to oral glucose administration in spite of higher fasting levels, while in the controls these levels increased dramatically (Figure 4D). Whole-body insulin sensitivity was assessed by performing insulin tolerance tests. ITT revealed that rats in the TD group had significantly less glucose in circulation after insulin treatment than the controls. Similarly, The AUC of ITT in the TD group was also significantly lower than that in the control and TD + TP groups (Figure 4C). We investigated whether testosterone deficiency induced an impairment of gluconeogenesis, which could contribute to the pathophysiological production and clearance of glucose. To this end, the gluconeogenic substrate precursor pyruvate was administered to the three rat groups (pyruvate tolerance test, PTT). The PTT measured the extension of the whole-body conversion of pyruvate into glucose and allowed for the assessment of the rate of gluconeogenesis, which was an important component of HGP. We found that the rats in the TD group had increased glucose production compared with controls (Figure 4B).

### Glucose appearance rates at the basal period

**Figure 5:**
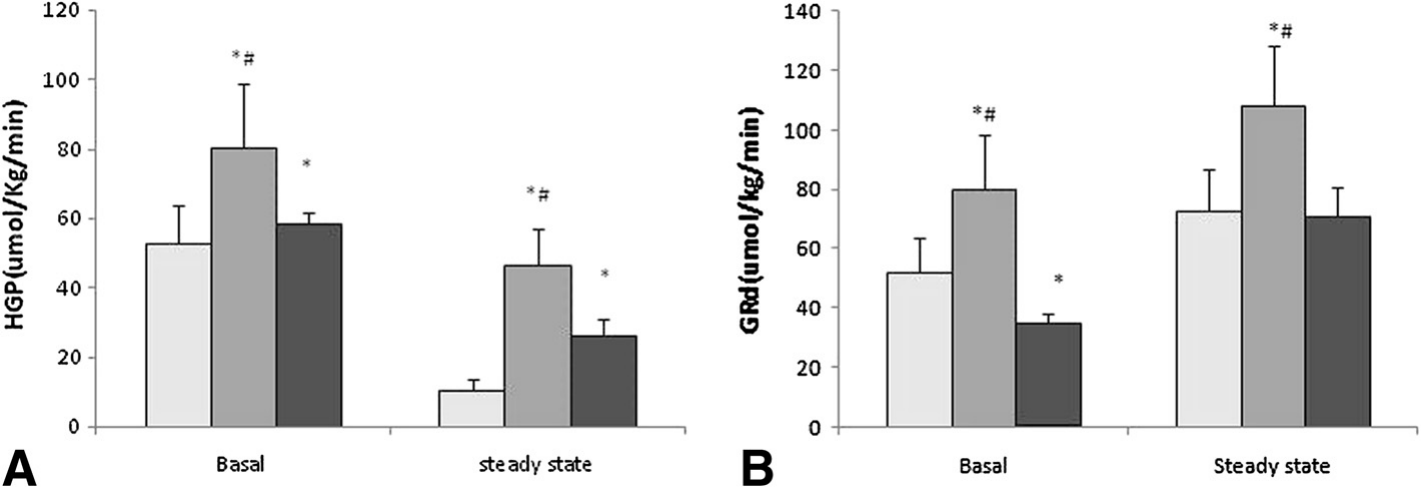
**HGP and the disappearance rate of glucose (GR**_**d**_**) in the basal state and hyperinsulinemic-euglycemic clamping steady-state. A**: HGP, **B**: GR_d_. *,P < 0.05, compared with the control group. ^#^, P < 0.05, compared with the TD + TP group.

After overnight fasting, the glucose appearance rates in the TD group were significantly higher compared with those in the control and TD + TP groups (TD group: 80.11+/−18.53 μmol/kg/min, control group: 52.30+/−11.37 μmol/kg/min, TD + TP group: 57.67+/−3.81 μmol/kg/min, P = 0.0087) (Figure 5A).

### Glycerol appearance and gluconeogenesis (GNG)

**Figure 6:**
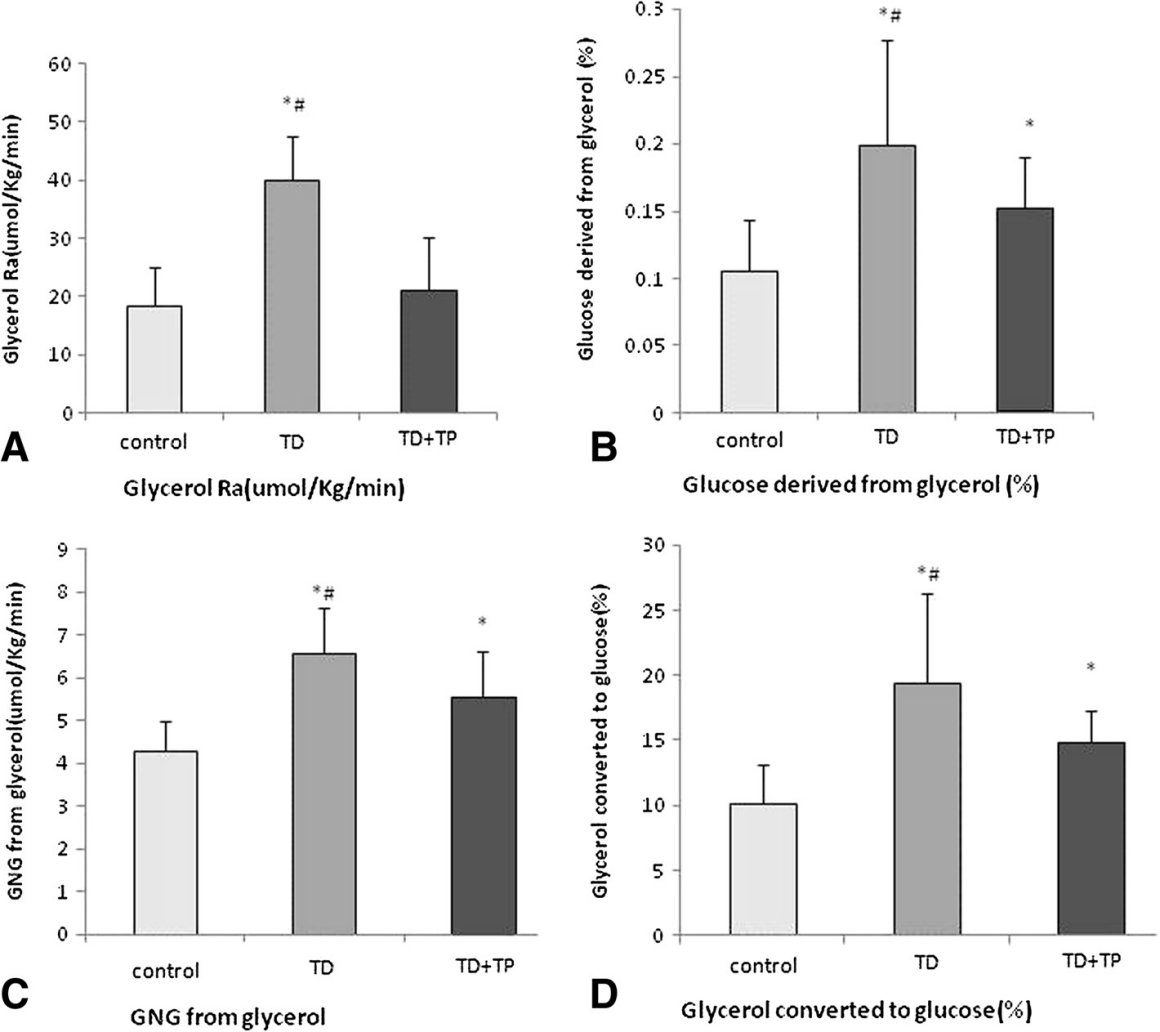
**Glycerol parameters in the three groups.** The glycerol appearance after an overnight fast was higher in the TD group compared with the control group and TD + TP group. **(A)** The parameters of gluconeogenesis from glycerol such as glycerol converted to glucose (%), glucose derived from glycerol (%) and gluconeogenesis from glycerol was higher (p < 0.05) in the TD group than in the control and TD + TP groups **(B, C, D)**. *, P < 0.05, compared with the control group. ^#^, P < 0.05, compared with the TD + TP group.

The glycerol appearance after overnight fasting was higher in the TD group than those in control group and TD + TP group (TD group: 39.99+/−7.70 μmol/kg^/^min, control group: 18.52+/− 6.72 μmol/kg^/^min, TD + TP group 21.26+/−9.00 μmol/kg^/^min, P = 0.0009) (Figure 6A). The parameters of the gluconeogenesis from glycerol including glycerol converted to glucose (%), glucose derived from glycerol (%) and gluconeogenesis from glycerol were remarkably higher in the TD group than those in the control group and TD + TP group (glucose derived from glycerol, TD: 19.96+/−7.74%, control: 10.52+/−3.75%, TD + TP: 15.23+/−3.81%, P < 0.05) (Figure 6B); gluconeogenesis from glycerol: (TD: 6.57+/−1.78 μmol.kg^-1^.min^-1^, control: 4.28+/−0.711 μmol.kg^-1^.min^-1^, TD + TP: 5.54+/−1.077 μmol.kg^-1^.min^-1^, P < 0.05) (Figure 6C); glycerol converted to glucose (%): (TD: 19.41+/−6.92%, control: 10.19+/−2.95%, TD + TP: 14.85+/−2.41%, P < 0.05) (Figure 6D).

### Concentrations of blood glucose and insulin at the clamp steady-state

**Figure 7:**
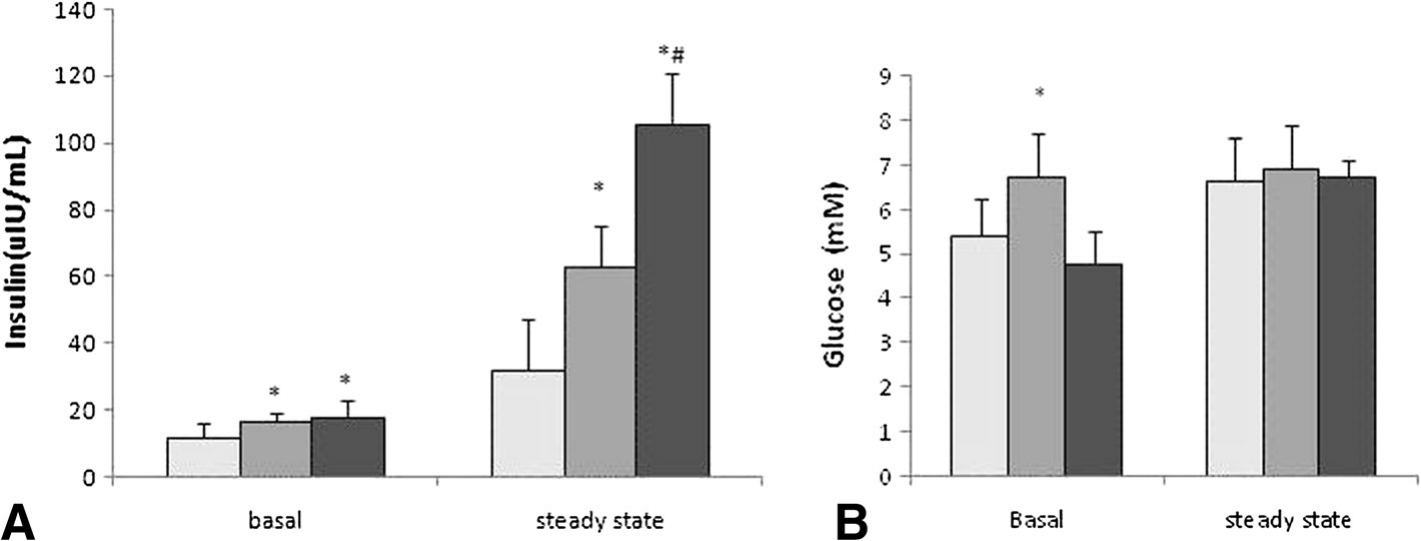
**Glucose and insulin concentrations in the basal state and hyperinsulinemic-euglycemic clamping steady-state. A**: insulin levels, **B**: glucose levels. *, P < 0.05, compared with the control group. ^#,^ P < 0.05, compared with the TD + TP group.

After exogenous insulin infusion (5 mIu/kg/min) for 90 minutes, the concentrations of blood glucose at clamp steady-state were similar among the three rat groups. Under basal conditions, the insulin concentrations in the TD group were significantly higher than those in the control group but lower than those in the TD + TP group (TD: 15.91+/−2.97 μIu/ml, control: 10.92+/−5.39 μIu/ml, TD + TP: 17.02+/−5.48 μIu/ml) (Figure 7B).

During the same period, the insulin concentrations of the three groups during the hyperinsulinemic-euglycemic clamp were dramatically elevated compared with their basal state. Similarly, the insulin concentration of the TD group was significantly higher than that of control group but lower than that in the TD + TP group (TD: 62.62+/−12.22 μIu/ml, control: 31.23+/−15.62 μIu/ml, TD + TP: 105.20+/−35.85 μIu/ml) (Figure 7A).

### Hepatic glucose production (HGP)

Despite higher insulin concentrations, the HGP following overnight fast remained higher in the TD group than in the control group (80.11+/−16.55 μmol/kg/min, 52.30+/−13.46 μmol/kg/min), implying an increased hepatic insulin resistance in the testosterone defective group. This finding was further confirmed during the exogenous insulin infusion when the endogenous insulin secretion was suppressed in all groups. Under these conditions, HGP remained greater (p < 0.01) in the TD group than in the control group (46.17+/−10.18 μmol/kg/min, 10.30+/−3.32 μmol/kg/min) (Figure 5A).

### Glucose disappearance rates at the clamp steady-state

After the exogenous insulin infusion (5 mIuU/kg/min) for 90 minutes, the glucose disappearance rate in the control group increased from 52.30+/−11.37 μmol/kg/min (the basal) to 72.49+/−14.12 μmol/kg/min (the steady state); in the TD group of rats, the glucose disappearance rate increased from 80.11+/−18.5 μmol/kg/min to 107.80+/−20.24 μmol/kg/min (p < 0.01), while in the TD + TP group, the glucose disappearance rate increased from 34.67+/−3.82 μmol/kg/min to 70.86+/−10.15 μmol/kg/min (P < 0.05). The growth rates of the three groups were 38%, 34% and 106%, respectively. Under the hyperinsulinemic-euglycemic clamp, the increment in the TD group was smaller than in the control and TD + TP groups (Figure 5B).

### Glucose infusion rates and Glucose uptake in muscle

**Figure 8:**
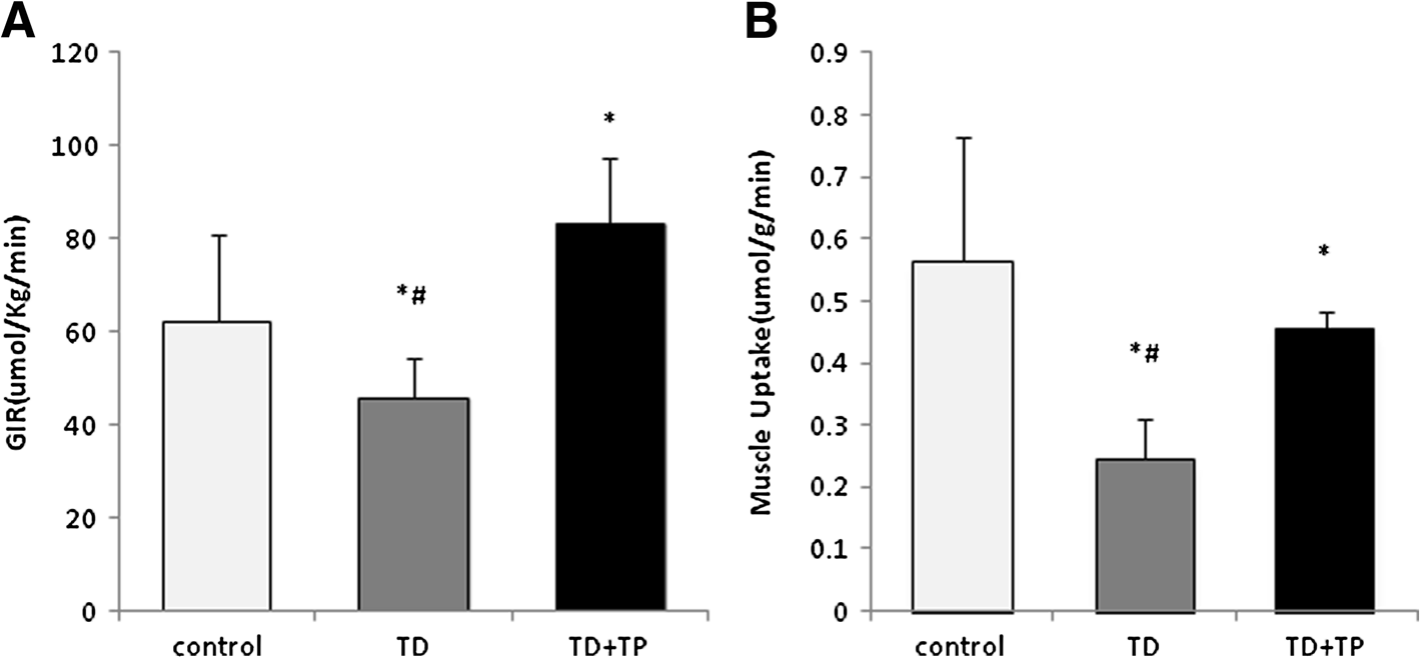
**Exogenous glucose infusion rates (GIR) and muscle glucose uptake during the hyperinsulinemic-euglycemic clamping steady-state. A**: GIR. **B** muscle glucose uptake during the hyperinsulinemic-euglycemic clamping steady-state. *,P < 0.05, compared with the control group. ^#^, P < 0.05, compared with the TD + TP group.

The exogenous glucose infusion rates required to maintain the glucose levels at the clamp point (6 ~ 7 mmol/L) were significantly reduced in the TD group; while in TD + TP group, they were higher than those in control group (TD: 45.69+/−8.73 μmol/kg/min, control: 62.306+/−18.55 μmol/kg/min, TD + TP: 82.94+/−14.46 μmol/kg/min), demonstrating insulin resistance in castrated rats (Figure 8A). The glucose uptake in the gastrocnemius was significantly lower in the TD group compared with that in the control and TD + TP groups (TD: 0.245+/−0.065 μmol/kg/min, control: 0.563+/−0.199 μmol/kg/min, TD + TP: 0.4574+/−0.025 μmol/kg/min, P < 0.05) (Figure 8B).

## Discussion

Testosterone changes body compositions and is positively correlated with lean mass and inversely correlated with fat mass in men. The effect that testosterone has on body weight varies between species. Smith et al. [13] reported that the mean body weight increased by 1.8% and fat mass by 11.0% after 12 months of ADT for patients with non-metastatic prostate cancer. The weight of the neutered male cats was 18.4% greater than the entire males over a period of 3 months and neutered dogs were found to be more likely to be overweight; however, castrated mice had less weight gain than uncastrated mice [14]. In this study, we found that rat body weight decreased after castration, and the final body weights were less than those of the control group. Testosterone reduction due to castration may have caused the weight loss due to loss of muscle and bone mass [15]. Despite a total weight loss, there was a visceral fat aggregation in the castrated rats. HE staining showed that the liver tissues were steatotic and that the muscle cells had intracellular fat in the castrated rats (pictures not shown).

Castrated rats are accepted model for studying the androgen deficiency in hypogonadal men [16]. As expected, the testosterone levels of the castrated rats in our study were significantly reduced while testosterone replacement therapy significantly raised the testosterone levels. Similar results were obtained by other studies whereby orchiectomy had caused 50% reduction in serum testosterone in male rats [17], while testosterone replacement was able to raise the testosterone levels [18].

Ten weeks after castration, we found that the baseline insulin concentrations in castrated rats were higher than those in normal rats, which conformed to the results in non-diabetic men [19,20]. However, glucose-stimulated insulin secretion while OGTT revealed that after glucose gavage, the increased insulin secretion rate was lower in the castrated rats. Moreover, the fasting glucose levels as well as the AUCs of PTT were slightly higher in the TD group, which indicated that there was a slight hepatic insulin resistance in the castrated rats. Interestingly, the OGTT in these rats were normal, and the ITT indicated that the castrated rats had increased their whole body insulin sensitivity. We think that this change was a compensatory process. These data suggested that in some stage of testosterone deficiency when glucose tolerance was normal while stimulated insulin secretion was undermined, the whole body insulin sensitivity slightly increased in order to maintain the normal blood glucose level. One possible mechanism for this might be hyperadiponectinemia according to the study of Nishizawa H et al. [21] who found that in rodents the serum testosterone level was inversely related to the serum adiponectin level, an insulin-sensitizing adipocyte-derived protein.

Insulin resistance (IR) can be central (hepatic) or peripheral (muscle, fat) depending on the primary site of involvement. Peripheral insulin resistance impairs glucose uptake from the blood into muscles (postabsorptive and fasting states), while hepatic IR manifests as unrestrained glucose production by the liver under fasting conditions [22]. HOMA-IR and ISI are frequently used to describe insulin sensitivity in many studies on testosterone deficiency. However, it is difficult to determine the primary site involved. The euglycemic-hyperinsulinemic clamp technique combined with isotope traces would assist in determining the source of the appearance and disappearance of plasma glucose metabolism. This combined technique could assist in accurately examining the metabolic flux of the carbohydrate by measuring the hepatic glucose production and glucose uptake in the periphery (to directly demonstrate insulin resistance).

Hepatic insulin resistance recently received increasing attention. Gluconeogenesis is a major source of fasting EGP, which maintains the body's fasting blood glucose at normal levels [23,24]. In diabetic patients, an increased gluconeogenesis from a substrate such as glycerol or alanine is the primary cause of hepatic insulin resistance. In this study, we used U-^13^C-glycerol to trace the naturally occurring metabolism kinetics of glycerol in the rat groups. Glycerol is a distinctive precursor of gluconeogenesis substrate, which often comes from peripheral triglyceride. We found that the glycerol appearance rates and gluconeogenesis (including glycerol converted to glucose (%) and gluconeogenesis from glycerol) were significantly higher in the castrated rats, while in castrated rats treated with testosterone propionate via intraperitoneal injection (25 mg/kg/day) for 30 weeks, they were reduced. At fasting, peripheral adipose tissue degradation is the primary source of glycerol. Due to the lack of glycerokinase in the adipose tissue, the glycerol released from it via lipolysis cannot resynthesize. Therefore, the glycerol appearance rate in the blood is a reliable indicator for lipolysis [25]. The high levels of glycerol appearance rates and gluconeogenesis in castrated rats suggested an increased lipolysis and hepatic insulin resistance. Our results also demonstrated significantly higher glucose appearance rates in the TD group compared with those in control rats. Thus, the absorption of glucose from the gastrointestinal system was limited when testosterone was deficient. Testosterone replacement therapy could partly relieve these metabolic disturbances.

Under hyperinsulinemic conditions, the HGP in the control group of rats at steady-state was nearly completely suppressed by insulin (suppressed to 19%); the castrated group showed a less dramatic decrease, only suppressed to 58%, which indicated hepatic insulin resistance. To maintain the clamp glucose level, the exogenous glucose infusion rates increased by nearly 27% in the control rats compared with those in the TD rats.

There is increasing awareness of the importance of muscle as a central player in adapting to excessive energy input (as it is one of the main fuel-consuming organs) [26]. Muscle accounts for a large proportion of metabolically active tissues of the body. Selective impairment of glucose disposal by muscle could contribute to the genesis of hyperglycemia and hence hyperinsulinemia. Approximately 80%-90% of exogenous glucose infusion is uptaken by the skeletal muscle [27], and it accounted for up to 70% of the whole body’s insulin sensitivity. In this study, we use carbon 14 labeled 2-deoxy-glucose to trace the glucose uptake rates of gastrocnemius under the clamp steady state. 2-deoxy-glucose has nearly the same feature as normal glucose, and it can be uptaken by animal tissues and phosphorylated to form 6-P-glucose in the cells without further metabolism. It is a good indicator for measuring the glucose uptake ability in target tissues as a classic method [28]. The average glucose uptake rates of gastrocnemius were lowest in the TD group. The replacement of testosterone propionate could partly increase the glucose uptake rates by gastrocnemius, but the rate could not reach the control levels.

In all, this study showed that castration-induced testosterone deficiency not only significantly enhances the hepatic gluconeogenesis but also significantly decreases extra-hepatic insulin sensitivity in advanced-aged male rats. The molecular mechanism how these occurred was still unidentified. Forkhead box protein O1 (FoxO1) has emerged as an important player in integrating insulin signaling to downstream target gene expression in carbohydrate metabolism. Moreover, Testosterone deficiency was commonly associated with conditions inducing muscle wasting. Akt signaling could control skeletal muscle mass through FoxO regulation of protein degradation, and this pathway have been previously identified as a target of androgen signaling. By immunohistochemical staining, we found that the FoxO1 protein expression increased and migrated into the nucleus in liver, skeletal muscle and pancreatic tissues in castrated rats (data were sorting for another paper).

## Conclusions

Castration-induced testosterone deficiency primarily affects fasting blood glucose and leads to increased levels of fasting glucose. Low testosterone levels not only significantly enhance the hepatic gluconeogenesis but also significantly decrease extra-hepatic insulin sensitivity in male rats.

## Abbreviations

AUC: Areas under the curve; E2: estrogen; EPG: Endogenous glucose production; FINS: Fasting plasma insulin; GC-MS: Gas Chromatography/Mass Spectrometry; HGP: Hepatic glucose production; IR: Insulin resistance; HOMA-IR: Homeostasis model-Insulin resistance; ISI: Insulin sensitivity index; ITT: Intraperitoneal insulin tolerance test; LOH: Late-onset hypogonadism; OGTT: Oral glucose tolerance test; PTT: Pyruvate tolerant test; Ra: the rate of glucose appearance; Rd: the rate of glucose disappearance; T: Testosterone; FoxO1: Forkhead box protein O1.

## Competing interests

The authors declare that they have no competing interests.

## Authors’ contributions

YL contributed study conceptualization, FX composed the manuscript, FX and XX performed the isotope infusion and clamp experiments, HZ and YM established the castrated animal model, HZ measured the hormone concentrations. All authors read and approved the final manuscript.
